# Editorial: Is it time to stick it to PFAS?

**DOI:** 10.3389/ftox.2023.1240563

**Published:** 2023-07-05

**Authors:** Ozge Cemiloglu Ulker, Priyanka Banerjee

**Affiliations:** ^1^ Department of Toxicology, Faculty of Pharmacy, Ankara University, Ankara, Türkiye; ^2^ Institute of Physiology, Charité University, Charité—Universitats Medizin Berlin, Corporate Member of Freie Universität Berlin, Humboldt-Universitat zu Berlin and Berlin Institute of Health, Berlin, Germany

**Keywords:** PFAS, hyperlipidemia, developmental toxicity, neurotoxicity, immune toxicity, one health

Since the 1950s, the usage of per- and poly-fluoroalkyl substances (PFAS), stable and complex compounds, in items like our clothes and kitchenware that we often use has increased. There are countless varieties of PFAS organic compounds, and due to their resistance to breakdown and extensive usage in industry and commerce, it has been discovered that they accumulate not only in our environment but also in our bodies. The high degree of possible exposure has given rise to specific concerns regarding PFAS. Its insidious encroachment into our life, from consuming PFAS-contaminated food and water to inhaling PFAS-contaminated air, is quite concerning. Additionally, it has been associated with a rise in blood pressure, a great variety of malignancies, developmental delays, and immune system disruption. It needs to be understood that if PFAS has adverse health effects notably related to long term chronic exposure.

The first article on this subject (Roth et al.) thoroughly analyses the health impacts of PFAS exposure sources. PFAS can enter the environment through production or waste streams and drinking water supplies. PFAS that are present in animals, food crops, and food packaging can also enter humans through their food and diets. PFAS can also migrate from products like carpets and clothing into the surrounding indoor air and dust. This study listed and concluded epidemiological data that connected PFAS exposure to metabolic disorders. Physiological endpoints and serum PFAS levels were displayed. The link between PFAS exposure in humans and hyperlipidemia and fatty liver disease has been shown in several research. The authors concluded that both long- and short-chain PFAS remain in drinking water sources globally based on previous human and animal studies and that regulators in nations all over the world may need to reexamine risk assessment and toxicity studies in order to evaluate the NOAEL and LOAEL for human populations accurately.

The second article on this subject (Abdulhasan et al.) is an original study that sought to test FUCCI ESCs for the effects of leading and lagging indicators of growth in a live imager and reported dose-dependent suppression of cell cycle commitment by PFOA and DEP as examples of the PFAS and phthalate families, respectively. A model involving embryonic and placental stem cells has shown that PFOA is distinct within the PFAS family in that it causes early first-trimester miscarriage. Additionally, it was noted that a temporal delay in the G1 phase prior to the S phase resulted in a green nadir in this unexpected FUCCI S-G2-M-phase report. The nadir quickly transforms into a green fluorescence peak by changing the medium. This research employed contact inhibition of growth in low stress-dose responders that achieve confluence at the earliest as a confounding variable and instead used toxicant dose-dependent suppression of this fed green peak as a sensitive indicator of future growth suppression.

A comprehensive evaluation and meta-analysis of the effects of PFAS on the brain and behaviour are presented in the third publication on this subject (Starnes et al.). Here, results from experimental investigations were compared with data from human epidemiological, experimental, and wildlife research, all of which show increased accumulation of perfluoroalkyl acids in the brain following environmental exposure. The results suggested the necessity for a more experimental investigation into the neurodevelopmental effects of PFAS concentrations and complex combinations that are relevant to the environment.

The fourth article on this subject (Guillette et al.) is an original study that is an illustration of a One Environmental Health study and aims to assess PFAS exposure and immune health biomarkers in populations of American alligators (*Alligator mississippiensis*). These protected species serves as a sentinel for the negative effects of persistent toxic pollutants. Increased PFAS exposure and immune system disruption may be linked to altered immune responses that cause autoimmune-like pathology in American alligators. This study supports and broadens the evidence that various PFAS are immune toxins seen in human epidemiological research and experimental models.

We hope that this Research Topic will contribute to providing new insights into the adverse health effects and environmental concerns of PFAS.

